# Association of genetic variants in the 3′UTR of HLA-G with Recurrent Pregnancy Loss

**DOI:** 10.1016/j.humimm.2016.06.020

**Published:** 2016-10

**Authors:** Giada Amodio, Valentina Canti, Luana Maggio, Susanna Rosa, Maria Teresa Castiglioni, Patrizia Rovere-Querini, Silvia Gregori

**Affiliations:** a San Raffaele Telethon Institute for Gene Therapy (SR-Tiget), Division of Regenerative Medicine, Stem Cells and Gene Therapy, IRCCS San Raffaele Scientific Institute, Via Olgettina 60, 20132 Milan, Italy; b Division of Immunology, Transplantation and Infectious Disease, IRCCS San Raffaele Scientific Institute, Via Olgettina 58, 20132 Milan, Italy; c Obstetric and Gynecology Unit, IRCCS San Raffaele Scientific Institute, Milan, Italy; d Vita-Salute San Raffaele University, Via Olgettina 60, 20132 Milan, Italy

**Keywords:** Unexplained Recurrent Pregnancy Loss (RPL), HLA-G, 3′UTR polymorphisms of *HLA-G*

## Abstract

Human Leukocyte Antigen (HLA)-G is involved in reprogramming immune responses at fetal-maternal interface during pregnancy. We evaluated the genetic diversity of the 3′ Un-Translated Region (UTR) of *HLA-G*, previously associated with *HLA-G* mRNA post-transcriptional regulation, in women with unexplained Recurrent Pregnancy Loss (RPL), with 2 pregnancy losses (RPL-2, n = 28), or 3 or more pregnancy losses (RPL-3, n = 24), and in 30 women with a history of successful pregnancy. Results showed in RPL-2, but not in RPL-3, women compared to controls: i) higher frequency of the 14 bp Ins allele, in single and in double copy; ii) significantly lower frequency of DelG/X genotype, iii) reduced frequency of the UTR-2, and UTR-3 haplotypes; iv) higher frequencies of the UTR-5, UTR-7, and UTR-8 haplotypes. This pilot study supports the relevance of performing 3′UTR *HLA-G* genetic screening, not limited to a specific polymorphism, but considering the extended haplotypes, as a possible predictor of pregnancy outcome.

## 1 Introduction

Sporadic miscarriage is the most frequent complication of early pregnancy. Approximately 70% of conceptions fail prior to live birth with most losses occurring prior to implantation or before the missed menstrual period [1]. Recurrent Pregnancy Loss (RPL) was initially defined as three or more consecutive pregnancy losses before 20–22 weeks of gestation [2]. Later, a large retrospective study indicated no differences in the frequency of diagnostic factors between women with two or more pregnancy losses [3]. Based on these results the Practice Committee of the American Society for Reproductive Medicine defined RPL as two or more failed pregnancies [4]. The prevalence of RPL is considerably lower than the prevalence of sporadic miscarriage, ranging from 0.8–1.4% to 2–3%, if biochemical losses (i.e. pregnancies that fail before ultrasound or histologic confirmation) are included [5]. Causes of RPL include chromosomal abnormalities, autoimmunity, such as untreated hypothyroidism and diabetes mellitus, uterine anatomic abnormalities, heritable thrombophilia, infections, and environmental cues [6]. The incidence of fetal chromosomal abnormalities, which is identified in 29–60% of women with RPL, decreases as the number of miscarriages increases, suggesting that other mechanisms may play a role in pregnancy loss [7]. The correction of endocrine disorders, and the treatment of the anti-phospholipid syndrome (APS), the most common autoimmune cause of pregnancy complications, have been shown to be effective in controlling miscarriage [6]. However, prevention of RPL remains an unmet medical need.

The uterus and the placenta constitute a unique site of immune modulation where the semi-allogeneic fetus is tolerated by the maternal immune system. Several years of research have identified the non-classical Human Leukocyte Antigen (HLA)-G, physiologically expressed on extravillous trophoblasts [8], as a molecule operatives in promoting tolerance during pregnancy [9]. HLA-G inhibits cytotoxic CD8^+^ T cells and natural killer (NK) cells, and allo-specific CD4^+^ T cell proliferation, modulates the activity of antigen presenting cells (APCs), and induces the differentiation of myeloid and T regulatory cells (reviewed in [10]). At the fetal maternal inferface during pregnancy the expression of HLA-G on trophoblasts prevents their NK-mediated lysis [11], and HLA-G-expressing regulatory T cells and APCs are highly represented [12], [13], supporting the critical role of HLA-G in promoting tolerance during pregnancy. In pregnancy complications, such as pre-eclampsia, it has been repetitively demonstrated a reduced levels of HLA-G expression in placental tissues [14], [15] and in the maternal circulation [15], [16], and defects in HLA-G-expressing regulatory cells [13]. Moreover, in RPL reduced levels of HLA-G have been reported [17].

The HLA-G *locus* contains several polymorphisms in the non-coding regions, including those present at the 3′Un-Translated Region (UTR), which influence HLA-G expression [18]. Among the 3′UTR of *HLA-G* polymorphisms, the most studied are an insertion/deletion (Ins/Del) of 14 base pairs (bp) that has been associated with *HLA-G* mRNA stability [19], [20], the G/C in position +3142 that can be involved in micro(mi)RNA-mediated post-transcriptional regulation [21], and the G/A at position +3187 that lands in an AU-rich region [22]. Other and less studied Single Nucleotide Polymorphisms (SNPs) are +3001 C/T, +3003 T/C, +3010 G/C, +3027 C/A, +3035 C/T, and +3196 C/G, which have been proposed as potential miRNA binding sites [23]. These polymorphisms arrange in haplotypes, named UTRs [18], and the most frequent are from UTR-1 to UTR-8 [24]. The association between 3′UTR haplotypes of *HLA-G* and protein expression has been recently studied: haplotypes containing 14 bp Del (i.e. UTR-1) are associated with high levels of soluble HLA-G, whereas those with 14 bp Ins (i.e. UTR-2, and UTR-5) with low HLA-G levels [25].

A lot of efforts have been made to define whether the genetic variations influencing the HLA-G expression can be associated with tolerance and/or pregnancy complications. Several studies have been conducted to define the influence of the 14 bp Ins/Del in recurrent miscarriage (RSA) with contrasting results [26], [27], [28]. Recent meta-analysis studies attributed the variability in the reported results to the heterogeneity of the cohorts analyzed and to the criteria used for patient selection [29], [30]. Nevertheless, Wang et al. [29] reported an association between the 14 bp Ins with recurrent miscarriage and an overall risk of RSA independently from the number of pregnancy losses, while Fan et al. [30] reported the same association only in women with three or more miscarriages. Thus far, the association between 3′UTR polymorphisms, not limited to the 14 bp Ins/Del, or the extended 3′UTR *HLA-G* haplotypes and RPL has been not investigated.

## 2 Subjects and methods

### 2.1 Study subjects

Subjects recruited in the study were admitted to the High-risk Pregnancy Outpatients Clinics of the San Raffaele University Hospital, Milan, Italy for investigation and treatment. The study sample included 52 patients diagnosed with RPL as two or more consecutive pregnancy losses confirmed by the Hospital records, according with the definition of the American Society for Reproductive Medicine [4]. Patients were subsequently divided in two different groups: women with 2 pregnancy losses (RPL-2, n = 28), and women with 3 or more pregnancy losses (RPL-3, n = 24). The control group comprised 30 age-matched women from couples with no history of miscarriages and a previous history of successful pregnancy (HP) (Table 1). Among the RPL group 28.8% had signs of autoimmunity, 15.4% had dysthyroidism, 1.9% had hypertension, and 17.3% had congenital thrombophilia, and none of them had uterine abnormalities as assessed by hysteroscopy or uterine ultrasonography. Conversely, 26.7% of HP had uterine anatomic abnormalities and after correction delivered successfully, and none of them was affected by other diseases. All RPL patients and their male partners had normal karyotypes. No others investigations on the male partners were performed. All patients were regularly menstruating and all had normal thyroxin levels in the plasma. Human peripheral blood was obtained upon informed consent in accordance with local ethical committee approval and with the Declaration of Helsinki.

### 2.2 DNA isolation

Genomic DNA was isolated from peripheral blood mononuclear cells (PBMCs), separated by density gradient centrifugation over Lymphoprep (Nycomed Amersham), using a commercial kit (QIAamp, QIAGEN, Italy) according to the manufacturer’ s instructions. The DNA samples were stored at −20 °C in a freezer compartment.

### 2.3 Amplification and sequencing of 3′UTR of the HLA-G gene (or genotyping)

100 ng of genomic DNA were amplified in a 25 μl reaction containing 1× polymerase chain reaction (PCR) buffer (Roche, USA), 0.2 mM dNTP mix (Roche, USA), 1.5 mM MgCl_2_ (Roche, USA), 0.8 U *Taq* Polymerase (Roche, USA), and 1 μM of each primer (For: 5′ TCACCCCTCACTGTGACTGA 3′; Rev: 5′ TTCTCATGTCTTCCATTTATTTTGTC 3′). The initial denaturation step was carried out at 95 °C for 3 min, followed by 30 cycles at 93 °C for 60 s, 58 °C for 60 s, 72 °C for 60 s, and by a final extension step at 72 °C for 10 min. The amplification product was evaluated using a 2.5% agarose gel, purified using a commercial kit (Wizard SV Gel and PCR Clean-Up System, Promega, WI, USA) according to the manufacturer’ s instructions. The Rev primer (5′ TTCTCATGTCTTCCATTTATTTTGTC 3′) by PrimmBiotech (Milan, Italy) was used to perform direct sequencing on both strands of purified amplification products. Sequencing pherograms were analyzed with the CodonCode Aligner software (Centerville, MA), and the 14 bp Ins/Del (rs1704), +3003 C/T (rs1707), +3010 C/G (rs1710), +3027 A/C (rs17179101), +3035 C/T (rs17179108), +3142 C/G (rs1063320), +3187 A/G (rs9380142) and +3196 C/G (rs1610696) polymorphic sites were individually annotated. Haplotype and genotype construction were assigned and named according to previous report [24] (Supplementary Table 1).

### 2.4 Statistical analysis

HLA-G 3′UTR allele and genotype frequencies were obtained by direct count. Single comparisons of allele and genotype frequencies between populations were performed using the Fisher test and, when necessary, Bonferroni correction for multiple comparisons was applied. Differences were regarded as significant at ^*^P < 0.05. The results were analyzed using GraphPad Prism 5.0 (GraphPad Software, Inc. La Jolla, CA).

## 3 Results and discussion

The main objective of this pilot study was to investigate the possible association of 3′UTR polymorphisms and haplotypes of *HLA-G* with RPL in a cohort of women with two or more pregnancy losses (RPL, n = 52). RPL subjects were subgroups in women with 2 pregnancy losses (RPL-2, n = 28) and women with 3 or more pregnancy losses (RPL-3, n = 24). As control we used a cohort of age-matched women from couples with no history of miscarriages (HP, n = 30) (Table 1).

We analyzed 8 out of the 16 polymorphisms previously described at the 3′UTR of *HLA-G* [18]: the 14 bp Ins/Del (rs1704), +3003 C/T (rs1707), +3010 C/G (rs1710), +3027 A/C (rs17179101), +3035 C/T (rs17179108), +3142 C/G (rs1063320), +3187 A/G (rs9380142) and +3196 C/G (rs1610696). Although no statistically significant differences were observed, we found that the 14 bp Ins allele that has been associated with low HLA-G protein levels [31] was more represented in RPL-2 compared to HP women both in single (50% vs. 35%) and in double (25% vs. 10%) copy (Table 2). Conversely, the 14 bp Del/Del genotype that has been associated with high levels of soluble HLA-G [32] was more represented in HP compared to RPL-2 women (37% vs. 25%; Table 2). No major differences were observed in the frequencies of the 14 bp Ins allele and of the 14 bp Ins/Ins genotype between RPL-3 and HP women (Table 2). Our data are partially consistent with those reported by Wang et al. [29], indicating the association of the 14 bp Ins with an increased risk of recurrent miscarriage. Conversely, the lack of association between 14 bp Ins with recurrent miscarriage in women with 3 or more pregnancy losses is in contrast to Fan et al. [30]. It cannot be excluded that this discrepancy can be attributed to the low number of women included in the present study.

As reported in Table 2, among other 3′UTR polymorphisms analyzed we observed a significant lower frequency of the +3035 C allele in RPL-2 compared to HP women both in single (84% vs. 97%, P = 0.0258) and in double copy (68% vs. 93%, P = 0.0190), but not between RPL-3 and the control group. Notably, the statistical significance was lost when we applied the Bonferroni correction (P = 0.2064 and P = 0.152, respectively).

The 14 bp polymorphism is in strong linkage disequilibrium with the +3142 C/G SNP that can be involved in HLA-G post-transcriptional regulation being the target of miR-148a, miR-148b, and miR-152 [21]. Of note, 14 bp Ins is always associated with +3142 G [33]. We therefore evaluated the genetic frequencies of the following combinations: InsG/InsG, DelC/DelC, InsG/DelC, and DelG/X. Results showed a higher, but not statistically significant, frequency of the InsG/InsG genotype in the RPL-2 compared to HP women (25% vs. 10%, P = 0.1729; Table 3). Moreover, a significantly lower frequency of DelG/X genotype was detected in RPL-2 compared to HP women (11% vs. 43.5%, P = 0.0078, and P = 0.0312 with Bonferroni correction; Table 3). Similarly, the frequency of the DelG/X genotype in the RPL-3 group was lower, but not statistical significant, compared to HP women (29% vs. 43.5%). No differences in the frequencies of DelC/DelC and InsG/DelC were observed neither in the RPL-2 nor in the RPL-3 compared to the HP (Table 3).

We next investigated the proportion of the most frequent extended 3′UTR haplotypes of *HLA-G* (from UTR-1 to UTR-8) in RPL and HP women. In line with previous data [18], [34], UTR-1 and UTR-2 resulted to be the most represented haplotypes in our cohorts of women (Table 4). UTR-3 (Del/+3003T/+3010C/+3027C/+3035C/+3142G/3187A/+3196G), which has been associated with intermediate/high levels of soluble or membrane-bound HLA-G [25], [35], was significantly less frequent in RPL-2 compared to HP women (P = 0.0078), but by applying the Bonferroni correction, the statistical significance was lost (P = 0.0624). Although no statistically significant differences were observed, a higher frequency of UTR-5 (Ins/+3003T/+3010C/+3027C/+3035T/+3142G/3187A/+3196C), UTR-7 (Ins/+3003T/+3010C/+3027A/+3035T/+3142G/3187A/+3196C), and UTR-8 (Ins/+3003T/+3010G/+3027C/+3035C/+3142G/3187A/+3196G) were observed in both RPL-2 and RPL-3 compared to women with successful pregnancy (Table 4). We next subdivided haplotypes into two sub-groups according to the presence of the 14 bp Ins (UTR-2, -5, -7 and -8) or Del (UTR-1, -3, -4 and -6) (Table 5). Among the 14 bp Del haplotypes, UTR-3 was significantly less represented in RPL-2 compared to HP women (P = 0.0240) (Table 5). Within the 14 bp Ins haplotypes, a significantly lower frequency of UTR-2 compared to UTR-5, UTR-7, and UTR-8 was found in RPL-2 compared to HP women (46% vs. 76%, P = 0.0448), but by applying the Bonferroni correction, the statistical significance was lost. This result is particularly relevant considering that, as previously indicated, the UTR-2 is the most represented haplotype in the worldwide population, whereas UTR-5, UTR-7, and UTR-8 haplotypes are normally found at low frequencies [18], [34]. The UTR-2, UTR-5, UTR-7, and UTR-8 have in common 14 bp Ins, +3142G and +3187A, which have been individually associated with low *HLA-G* mRNA levels [36]. However, they differ in +3010 C/G, +3027 C/A, +3035 C/T, and +3196 C/G, suggesting that these polymorphisms may be also important in modulating the expression levels of HLA-G. In line with this hypothesis are results in which the presence of UTR-5 and UTR-7 haplotypes has been associated with low levels of soluble HLA-G, whereas UTR-2 with intermediate levels [25].

In conclusion, in this pilot study we analyzed for the first time the extended 3′UTR *HLA-G* haplotypes in women experienced RPL. Our results suggested an association of specific 3′UTR haplotypes of *HLA-G* with 2 consecutive, but not with 3 or more, pregnancy losses, although the statistical significance was not always reached. We cannot exclude that enlarging the cohorts of women we may strengthen the associations. Although more comprehensive study, in which the evaluation of other HLA-G gene segments, such as the promoter and the coding region, may help in the study of the expression of HLA-G, this study confirms the important role of HLA-G in pregnancy. Moreover, these findings support the relevance and foster the importance of performing 3′UTR *HLA-G* genetic screening, not limited to a specific polymorphism, but considering the extended haplotypes, as a predictor of pregnancy outcome.

## Authorship contributions

G.A. and V.C. performed the experiments, collected and analyzed data and wrote the manuscript; L.M. collected samples; S.R. and M.T.C. enrolled patients; P.R.-Q. and S.G. conceived the scientific idea, supervised the project, and wrote the manuscript.

## Conflict of interest

The authors declare that there is no conflict of interests regarding the publication of this paper.

## Figures and Tables

**Table 1:** Patients characteristics and pregnancy history.

	RPL-2 women	RPL-3 women	HP women
Patients (N)	28/52	24/52	30
Age (mean ± SD)	36.3 ± 4.3	36.5 ± 4.3	36.1 ± 4.0
Caucasian (N, %)	26/28 (92.8)	22/24 (91.7)	27 (90)
Miscarriages (N, %)	57/69 (82.6)	92/112 (82.1)	0
Intrauterine fetal death (N, %)	1/69 (1.4)	1/112 (0.9)	0
Live birth (N, %)	11/69 (16)	19/112 (17)	38 (100)

**Table 2:** Estimated allele and genotype frequencies observed at *HLA-G* 3′UTR polymorphic sites in RPL and HP women.

Polymorphisms	RPL-2 (N = 28)	Freq (%)	RPL-3 (N = 24)	Freq (%)	HP (N = 30)	Freq (%)	*P^2^*	*P^3^*
*14 bp*								
Ins	28	50	20	44	21	35	0.1327	0.5511
Del	28	50	28	56	39	65		
Ins/Ins	7	25	4	17	3	10	0.1729	0.6868
Del/Del	7	25	7	29	11	37	0.4022	0.7720
Ins/Del	14	50	13	54	16	53	1.0000	1.0000

*+3003*								
C	10	18	6	12.5	6	10	0.2843	0.7629
T	46	82	42	87.5	54	90		
C/C	0	0	1	4	1	3	1.0000	1.0000
T/T	18	64	19	79	25	84	0.1362	0.7363
C/T	10	36	4	17	4	13	0.0667	1.0000

*+3010*								
C	26	46.5	22	46	32	53	0.5774	0.5615
G	30	53.5	26	54	28	47		
C/C	8	28	7	29	8	27	1.0000	1.0000
G/G	10	36	9	37.5	7	23	0.3902	0.3695
C/G	10	36	8	33.5	15	50	0.3012	0.2738

*+3027*								
A	4	7	2	4	1	2	0.1953	0.5838
C	52	93	46	96	59	98		
A/A	0	0	0	0	0	0	–	–
C/C	24	86	22	92	29	97	0.1866	0.5791
A/C	4	14	2	8	1	3	0.1866	0.5791

*+3035*								
C	47	84	44	92	58	97	**0.0258^∗^**	0.4032
T	9	16	4	8	2	3		
C/C	19	68	20	83	28	93	**0.0190^∗^**	0.3890
T/T	0	0	0		0	0	**–**	–
C/T	9	32	4	17	2	7	**0.0190^∗^**	0.3890

*+3142*								
C	25	45	20	42	25	42	0.8515	1.0000
G	31	55	28	58	35	58		
C/C	5	19	4	17	3	10	0.4637	0.6868
G/G	8	28	8	33	8	27	1.0000	0.7653
C/G	15	53	12	50	19	63	0.5945	0.4098

*+3187*								
A	42	75	35	73	42	70	0.6781	0.8317
G	14	25	13	27	18	30		
A/A	14	50	11	46	14	46.5	1.0000	1.0000
G/G	0	0	0	0	2	7	0.4918	0.4969
A/G	14	50	13	54	14	46.5	1.0000	0.7846

*+3196*								
C	38	68	34	71	43	72	0.6896	1.0000
G	18	32	14	29	17	28		
C/C	12	43	11	46	14	46.5	0.7976	1.0000
G/G	2	7	1	4	2	7	1.0000	1.0000
C/G	14	50	12	50	14	46.5	1.0000	1.0000

*P^2^* = Comparison between RPL with 2 pregnancy losses (RPL-2) and HP using Fisher’s test.

*P^3^* = Comparison between RPL with 3 or more pregnancy losses (RPL-3) and HP using Fisher’s test.

∗ = Statistical significance was lost after Bonferroni correction for multiple comparisons.

Bold values are significant values obtained comparing RPL-2 versus HP women.

**Table 3:** Estimated genotype frequencies observed at *HLA-*G 3′UTR polymorphic sites in RPL and HP women.

Polymorphisms	RPL-2 (N = 28)	Freq (%)	RPL-3 (N = 24)	Freq (%)	HP (N = 30)	Freq (%)	*P^2^*	*P^3^*
*14 bp/+3142*								
InsG/InsG	7	25	4	17	3	10	0.1729	0.6868
DelC/DelC	5	18	5	21	4	13	0.7260	0.4542
InsG/DelC	13	46	8	33	10	33.5	0.4214	0.7654
DelG/X	3	11	7	29	13	43.5	**0.0078/0.0312^∗^**	0.7688

*P^2^* = Comparison between RPL with 2 pregnancy losses (RPL-2) and HP using Fisher’s test.

*P^3^* = Comparison between RPL with 3 or more pregnancy losses (RPL-2) and HP using Fisher’s test.

∗ = Statistical significance calculated with/without Bonferroni correction for multiple comparisons.

Bold values are significant values obtained comparing RPL-2 versus HP women.

**Table 4:** Estimated haplotype frequencies observed at *HLA-*G 3′UTR polymorphic sites in RPL and HP women.

Haplotypes	RPL-2 (N = 28)	Freq (%)	RPL-3 (N = 24)	Freq (%)	HP (N = 30)	Freq (%)	*P^2^*	*P^3^*
UTR-1	14	25	13	27	18	30	0.6781	0.8317
UTR-2	13	23	9	19	16	27.5	0.8304	0.3671
UTR-3	3	5	7	15	14	23	**0.0078^∗^**	0.3300
UTR-4	9	16	5	10.5	6	10	0.4108	1.0000
UTR-5	5	9	3	6	1	1.5	0.1051	0.3209
UTR-6	2	4	3	6	1	1.5	0.6089	0.3209
UTR-7	4	7	3	6	1	1.5	0.1953	0.3209
UTR-8	6	11	5	10.5	3	5	0.3105	0.4621

*P^2^* = Comparison between RPL with 2 pregnancy losses (RPL-2) and HP using Fisher’s test.

*P^3^* = Comparison between RPL with 3 or more pregnancy losses (RPL-3) and HP using Fisher’s test.

∗ = Statistical significance was lost after Bonferroni correction for multiple comparisons.

Bold values are significant values obtained comparing RPL-2 versus HP women.

**Table 5:** Estimated allelic frequencies of 3′UTR haplotypes of *HLA-G* in RPL and HP women.

	Haplotypes	RPL-2 (N = 28)	Freq (%)	RPL-3 (N = 24)	Freq (%)	HP (N = 30)	Freq (%)	*P^2^*	*P^3^*
14 bp Del	UTR-1	14	50	13	46	18	46	0.8076	1.0000
	UTR-4-6	11	39	8	29	7	18	0.0920	0.3777
	UTR-3	3	11	7	25	14	36	**0.0240^∗^**	0.4276

14 bp Ins	UTR-2	13	46	9	45	16	76	**0.0448^∗^**	0.0578
	UTR-5-7-8	15	54	11	55	5	24	**0.0448^∗^**	0.0578

*P^2^* = Comparison between RPL with 2 pregnancy losses (RPL-2) and HP using Fisher’s test.

*P^3^* = Comparison between RPL with 3 or more pregnancy losses (RPL-3) and HP using Fisher’s test.

**∗** = Statistical significance was lost after Bonferroni correction for multiple comparisons.

Bold values are significant values obtained comparing RPL-2 versus HP women.

## Appendix A Supplementary data

Supplementary Table 1 3′UTR HLA-G haplotypes.
