# Supplementary material for: Association of genetic variants in the 3′UTR of HLA-G with Recurrent Pregnancy Loss
Source: Hum Immunol. 2016 Oct;77(10):886–91. doi: 10.1016/j.humimm.2016.06.020 (PMC5021086; doi:10.1016/j.humimm.2016.06.020)
Supplement: Supplementary Table 1 — 3′UTR HLA-G haplotypes. [file mmc1.docx]

| Haplotypes | Polymorphisms | | | | | | | |
| --- | --- | --- | --- | --- | --- | --- | --- | --- |
|  | **14bp** | **+3003** | **+3010** | **+3027** | **+3035** | **+3142** | **+3187** | **+3196** |
| UTR-1 | **Del** | T | G | C | C | C | G | C |
| UTR-2 | Ins | T | C | C | C | G | A | C |
| UTR-3 | Del | T | C | C | C | G | A | G |
| UTR-4 | Del | C | G | C | C | C | A | C |
| UTR-5 | Ins | T | C | C | T | G | A | C |
| UTR-6 | Del | T | G | C | C | C | A | C |
| UTR-7 | Ins | T | C | A | T | G | A | C |
| UTR-8 | Ins | T | G | C | C | G | A | G |

**Supplementary Table 1. 3’UTR HLA-G haplotypes**
